# Emerging evidence-based physical rehabilitation for Multiple Sclerosis - Towards an inventory of current content across Europe

**DOI:** 10.1186/1477-7525-8-76

**Published:** 2010-07-28

**Authors:** Kamila Rasova, Peter Feys, Thomas Henze, Hans van Tongeren, Davide Cattaneo, Johanna Jonsdottir, Alena Herbenova

**Affiliations:** 1 Department of Rehabilitation, Third Medical Faculty, Charles University, Ruská 87, 100 00 Prague 10, Czech Republic; 2 REVAL Rehabilitation & Healthcare Research Center, PHL-University College and BIOMED, University of Hasselt, Belgium; 3 Reha-Zentrum Nittenau, Germany; 4 Sclerosecenter in Haslev, Denmark; 5 Don Gnocchi Foundation, Milano, Italy

## Abstract

In Europe, theoretical approaches to physical therapy and rehabilitation in multiple sclerosis often appear significantly different. While there is general agreement that rehabilitation plays an important role in maintaining and improving function in persons with multiple sclerosis, no consensus exists on what may be the most effective approach to achieve the best possible functionality within an individual's limitations.

The objective of this paper is to initiate an analysis of currently applied physical interventions for people with multiple sclerosis throughout Europe during inpatient or outpatient rehabilitation programs. A study of the content of rehabilitation may show presently performed treatment methods revealing the basic considerations that nowadays guide clinicians implicitly or explicitly in the treatment of persons with multiple sclerosis. Following this first step, comparative studies can be set up.

## Introduction

Neurological abnormalities due to multiple sclerosis (MS) manifest themselves with a wide range of symptoms like fatigue, numbness, paraesthesias, muscular weakness and spasticity, double vision, optic neuritis, ataxia, bladder control problems, dysphagia, dysarthria and cognitive dysfunction. These impairments can lead to relevant problems in carrying out activities of daily living, and participation, too. Therefore, rehabilitation is focused on functional disabilities evolving from the reported symptoms such as balance disorders, gait abnormalities, etc. Symptoms of MS are of different severity and thus cause different problems in every stage of the disease [1-3].

There is no curative treatment available for MS yet. Despite the fact that drug-induced immunosuppression and immunomodulation have been shown to decelerate the inflammatory-related progression of MS [4-7], there are numerous symptoms (such as fatigue, pain, spasticity, bladder dysfunction) and considerable disabilities (such as reduction of mobility, communication, and cognitive function) that develop during the course of the disease and require specific symptomatic treatments [8]. Symptomatic treatment [2] does not include only drugs but additionally a large body of functional interventions, especially physical treatment methods, occupational, speech and swallowing therapy, as well as neuropsychological training, which are all important parts of comprehensive rehabilitation programs. Symptomatic drug treatment and rehabilitation are both recommended to stabilise or improve the functional status of person with multiple sclerosis (PwMS).

## Discussion

### Empirical evidence for a basis of rehabilitation

The processes that lead to functional recovery after rehabilitation have been intensively debated within the last years. Rehabilitation in general "has moved from professional artistry to an evidence-based scientific approach over the last 15-20 years". This very clear and refined statement by D. Richardson [9] is true for MS rehabilitation, too. In the past, MS rehabilitation was performed only rarely and non-systematically but is now steadily maturing and being attributed more importance due to the following observations:

⇨ **A growing body of neuroscientific knowledge on fundamental aspects underlying rehabilitation has emerged including**:

• growing knowledge about neuroplasticity and the ability of the central nervous system to trigger and/or promote reorganization of damaged structures and function,

• better understanding of the biochemical factors that promote learning and neural remodelling (neurotrophic factors, neurotransmitters, etc.) in a close relationship with the activation or reactivation of neural cell precursors, responsible for reparative processes,

• advances in the understanding of neuropsychological factors, such as the systems of memory, executive function and attention (all of which are cognitive functions often altered in MS patients); increasing knowledge of motor control and motor learning [10,11].

⇨ **Rehabilitation in general as well as specific physical interventions have been shown to be effective**. In the last decade the number, as well as the quality of published scientific studies and systematic reviews in MS rehabilitation have clearly increased [3,12]. Studies have revealed short- and long-term beneficial effects of comprehensive rehabilitation programs (multimodal rehabilitation) [12-15] also studies of physical interventions, including exercise therapy, have demonstrated their effectiveness [16-20].

⇨ **Contextual changes in the rehabilitation field revealing**

• an understanding of the importance of properly classifying and recognizing the different health problems that a patient is confronted with, using the International Classification of Functioning, Disability and Health (ICF), a globally-agreed-upon framework of the World Health Organization. It was recently recommended that clinical practice in MS, including rehabilitation, should be based on this classification system which relates the typical spectrum of problems in functioning of PwMS with their personal attitudes and the environmental context in which they live [21]. This approach is essential for assessment and selection of the best strategies for rehabilitation [22,23],

• the awareness of the necessity of implementing evidence-based knowledge and practice (patient's values, therapist's experience, and scientific evidence) into rehabilitation,

• an increasing interest in Health-related Quality of Life as an essential outcome measure for treatment [24,25].

⇨ **Increased political attention towards high-quality rehabilitation for MS and equal chances of access**. Rehabilitation in chronically disabled people like PwMS has gained growing attention. This is one of the reasons why the European MS Platform (EMSP) developed and published the "Code of Good Practice" claiming that all PwMS throughout Europe should have "equal rights and access to treatment, therapies and services in the management of Multiple Sclerosis" [26]. This document has been endorsed by the European Parliament, and as such, rehabilitation in MS has been more widely accepted. Nowadays, it is important to work on the political and legislative implementation of the Code of Good Practice of Rehabilitation (early, long term, aimed, comprehensive, and attainable for everybody) across Europe, and ensure access to care.

### Drawbacks in current practice and research

First, MS is an individually variable and unpredictable disease needing evaluation at different assessment levels (impairment, disability, handicap, quality of life), including patient-reported outcome measures. Unfortunately, both researchers and therapists currently use too many different measures or do not address all levels of patient/therapist perspectives, thus making it difficult to directly compare the effectiveness of interventions (about 2600 articles have been found in PubMed about outcome measures in MS). Interestingly, regional differences in using specific outcome measures in MS across Europe were reported in a study of Haigh et al. [27]. The Authors found some variation in the preference for specific measures across Europe. The differing choice between competing instruments, such as the Functional Independence Measure versus the Barthel Index, was likely related to the specific contexts in different regions rather than discussion on the need of the domains to be measured. A surprising finding was the low level of use of the so-called 'generic measures' in routine clinical practice. At the XIII. SIG Mobility of RIMS (Rehabilitation in Multiple Sclerosis, the European network of MS centres) meeting "Content of physical rehabilitation in multiple sclerosis" 2010, different approaches to evaluation were presented, for example an application of the ICF in documenting the term limitations of the MS disease, goal attainment scaling (GAS) - a method for rating goal achievement, and Patient Reported Outcomes Measurement Information Systems (PROMIS) - recently used modern psychometric Theory. The use of each approach probably depends on social and health policy system in each country [28]. The role of policy systems in disparities within Europe is mentioned in a very recent study [29] that compared treatment and care of MS in chosen six countries with different geography, culture, and economical and politic systems. Prodiner et al. 2010 [30] confirmed that policy factors influence participation in work or social life and recommended the development of participation outcome measurements. No study until now has evaluated the impact of socio-political environment on the choice of outcome measures within Europe, an issue that should be studied in the future. In line with Kwakkel et al. [31], we advocate to reach for a world-wide consensus on the use of outcome measures in MS rather than development of new ones. In this regard, the ICF 'core set' for MS should be considered [22]. We also encourage current collaborative activities within RIMS and CMSC, the Consortium of Multiple Sclerosis Centers, to reach consensus on gait and fatigue outcome measures for MS [32]. A common set of outcome measures will at a later stage facilitate comprehensive meta-analyses which could better reflect the true efficacy of rehabilitation in MS than individual studies with small sample sizes. Besides, it is likely that a modular evaluation approach is needed given the broad variety in severity of symptoms that may occur between and within patients [33].

Second, even though an increasing number of studies have been published in the field of physical rehabilitation (about 1000 articles were found in PubMed about physical rehabilitation in MS from 1960 s until now), there is still restricted conclusive scientific evidence for the efficacy of treatment interventions in MS. This is related not only to the use of different outcome measures or limited sample sizes, but also to the variety in patient characteristics (severity of symptoms, type of MS, age, subjective factors), treatment goals, treatment setting (in-patient versus out-patient care, multi-disciplinary versus isolated intervention), duration and intensity ("dosage") of treatment as well as time points of measurements (pre, post as well as follow-up) [3,12-15,18,19,34-38]. As a consequence, even with available studies regarding certain interventions, it remains sometimes unclear which interventions are effective at what stage of MS or at which level of disability, which leads to limited transfer of evidence into daily practice. We acknowledge that some of these factors are likely to be influenced by the local organization of rehabilitation but emphasize a better standardization when doing research.

Third, it is widely agreed that physical rehabilitation includes a variety of techniques and conceptual treatment methods that are not yet studied by rigorous scientific methods but nevertheless may be of value. For example the effectiveness of Vojta reflex locomotion, Proprioceptive neuromuscular facilitation or Perfetti concept and other generally known and accredited methods, has not been scientifically confirmed in MS (no publication was found in PubMed about the above mentioned methods in MS). Education, culture, history and the way of philosophical thinking (how the patient is being perceived); focus on symptomatic or facilitation or task-oriented intervention [39]) have led to different kinds of therapeutic approaches across Europe which will be briefly discussed below. We are convinced, that a main drawback in current clinical practice and research is that the precise content of interventions is often poorly documented (what were therapist and patient really doing during intervention?) while, on the other hand, different terminology may be used to address similar approaches. An inventory of current content of rehabilitation defined for different symptoms, symptom severity, functioning problems and treatment goals would facilitate exchange of therapeutic knowledge and a set-up of comparative studies in MS.

Fourth, different Health Systems and Policies (Funding Health Care, Human Resources for Health, Health Services Management, Health Economics, Health Technology Assessment, decentralization versus centralisation in Health Care, private versus public Medical Insurance, Social Health Insurance systems, Assuring quality of Health Care, Caring for People with Chronic Conditions, Primary Care, Disease Prevention, etc.) have an impact on rehabilitation and physiotherapy approaches and methods. Also the educational systems and highest levels in physiotherapy (professional bachelors versus academic masters) are still different in European countries including the access to postgraduate education [40,41]. This topic has not been mapped in MS yet.

Fifth, the gap between what is known about effective health services and what is done in real-world practice exists. Deficiencies in the adoption of new strategies and findings in clinical practice were found [42]. It is important to understand how information from research studies and non-evidence-based opinions (opinion from clinical expert leaders, universities, consumers, direct service providers etc.) is transferred to the clinical field and to what extent it may be the barrier that hampers the transfer of new knowledge [43,44]. Cabana et al., 1999 [43] categorized types of the barriers: lack of awareness (the difficulty to be aware of every applicable approach and critically apply it to practice), lack of familiarity with new evidence, lack of agreement with new approaches, lack of self-efficacy (the belief that one can actually perform a change in clinical practice), lack of outcome expectancy on new methods, inertia of previous practice of clinicians that may not have the motivation to change, patient-related barriers (the inability to reconcile patient preferences with recommendations and environmental-related barriers that address the acquisition of new resources or facilities). Dissemination for outcome measures and description of new approaches must overcome these barriers [43]. Besides standard possibilities of information dissemination like using print information materials (leaflets, posters), mass media including internet, shows and exhibitions, scientific publications or papers, there are more effective techniques like self-directed curricula and small group interactions that help learners assess the discrepancy between what they ought to know or do and what they know or do, and provide opportunities to try out an innovation before putting it into practice [43]. However these techniques are time and cost consuming, too dependent upon local health organizations and on translation techniques that can be problematic (e.g. translation to national language, back-translation to English, verification of mismatch, again translation into national language, translation by different persons). New methods based on innovative technological models could provide a system to exchange information at lower costs and with a wider spectrum of users. Only few articles about dissemination information, of which none on the MS disorder, have been written. Research on dissemination of information (how information about health care interventions are created, packaged, transmitted, and interpreted among a variety of important stakeholder groups) is indicated in order to effectively facilitate transfer of knowledge to evidence-based interventions.

### Historical description of physical rehabilitation

In the last 60 years a great variety of techniques and conceptual treatment methods have been proposed and applied in the clinical field. Some methods have already been used since the 1950 s (for example, the Bobath concept, proprioceptive neuromuscular facilitation, Vojta reflex locomotion) and are still in use [45]. Originally their theoretical approaches were based on the hierarchic model of motor control and were applied in physiotherapy as so called facilitation approaches. However, with the development of sophisticated imaging methods like functional magnetic resonance imaging and subsequently increasing knowledge on neuroplasticity and its prerequisites, the hierarchical model of motor control has been contested. A more recent model of motor control is the systems model [46] which forms the basis for the task-oriented therapeutic approach, or in a wider concept, problem solving approach, focusing on specific disabilities of an individual patient. The application of some "older" methods has changed in this direction and new methods have been developed (for example motor relearning programs).

All methods have in common that they apply internal and external stimuli to achieve better movement, with the aim of improving activities of daily living. The facilitation approach puts the accent on manual application of stimuli (by proprioceptive and exteroceptive stimulation, in Bobath concept e.g. by so called handling, in Vojta reflex locomotion by stimulating of so called initiation zones in precisely-defined positions) with the aim to facilitate and improve a given motor function, movement pattern or to start a locomotion program, while the quality of execution is carefully controlled [47]. The task-oriented approach makes use of mainly behavioral requests and a patient learns by repeating a given specific task in different environments/under different conditions. The ability to carry out a specific task may be more important than the quality of the execution [48,49]. It can be argued that task-oriented approach draws on or is close to the ICF system [22,23,50] in that it considers recovery at the activity level.

### Theoretical bases of current clinical practice

Three main (physio-)therapeutic approaches based on models of motor control are being used and discussed nowadays [51]:

▪ muscle re-education, e.g. bio-feedback, aerobic training, and muscle strengthening,

▪ neurotherapeutic facilitation, e.g. Vojta reflex locomotion, Brunnström, Rood, Bobath, proprioceptive neuromuscular facilitation, and the

▪ task-oriented approach, e.g. Petö concept, Constraint-Induced Movement Therapy, Motor Relearning Programme, "contemporary" (modified) Bobath concept, locomotor training and Dual Tasking methods.

The theoretical bases of the different models are partially overlapping and cannot in all cases be strictly separated from each other. In the physiotherapeutic practice it is usually very difficult to define the approach used. Physiotherapists are led by their clinical experience and intuition on the one hand and their knowledge of evidence based medicine on the other. They sometimes combine different therapeutic approaches based on different theoretical models, everybody with the same aim to ameliorate functionality, participation and well-being of PwMS. Nevertheless, it is obvious that the interventions differ not only in content and terminology used, but also in their definitions of content of treatment, partial aims and understanding of therapeutic principles.

## Conclusion

A Special Interest Group on Mobility, part of RIMS http://www.rims.be aims at starting an inventory of content of physical rehabilitation both on the level of (i) therapeutic content/philosophy/terminology and (ii) documentation of organisation of care (intensity and location of treatment, clinicians involved in the process etc).

The inventory may be not only a stock-check of the actual MS rehabilitation practice across Europe but may also serve as a basis for further (comparative) scientific work to put MS- rehabilitation on a higher and more widely applied level and so fulfil some of the demands of the Code of Good Practice (to impact patients' quality of life, local and national health organizations, and health insurance companies).

## Competing interests

The authors declare that they have no competing interests.

## Authors' contributions

Each author has participated sufficiently in the work to take public responsibility for appropriate portions of the content. All authors have read the final manuscript.
